# Predictors of PSMA PET Positivity: Analysis in a Selected Cohort of Biochemical Recurrence Prostate Cancer Patients after Radical Prostatectomy

**DOI:** 10.3390/cancers15184589

**Published:** 2023-09-15

**Authors:** Paola Mapelli, Samuele Ghezzo, Cristiano Pini, Ana Maria Samanes Gajate, Alessandro Spataro, Carolina Bezzi, Claudio Landoni, Paola Scifo, Alberto Briganti, Arturo Chiti, Maria Picchio

**Affiliations:** 1 Faculty of Medicine and Surgery, Vita-Salute San Raffaele University, 20132 Milan, Italy; mapelli.paola@hsr.it (P.M.); ghezzo.samuele@hsr.it (S.G.); bezzi.carolina@hsr.it (C.B.); briganti.alberto@hsr.it (A.B.); chiti.arturo@hsr.it (A.C.); 2 Nuclear Medicine Department, IRCCS San Raffaele Scientific Institute, 20132 Milan, Italy; samanesgajate.anamaria@hsr.it (A.M.S.G.); scifo.paola@hsr.it (P.S.); 3 School of Medicine and Surgery, University of Milano-Bicocca, 20126 Milan, Italy; pini.cristiano@hsr.it (C.P.); alessandro.spataro@outlook.it (A.S.); claudio.landoni@unimib.it (C.L.); 4 Department of Urology, Division of Experimental Oncology, Urological Research Institute (URI), IRCCS San Raffaele Scientific Institute, 20132 Milan, Italy

**Keywords:** prostate cancer, biochemical recurrence, PSMA, PET

## Abstract

**Simple Summary:**

Localized prostate cancer can be treated with radical intent via surgery, yet up to 50% of patients experience a rise in prostate-specific antigen (PSA) serum levels during the post-surgical follow-up, a condition defined as a biochemical recurrence that requires additional examinations and treatments. PSMA PET is the most accurate diagnostic tool to detect disease recurrence in patients with biochemical recurrence. In the present work, a retrospective analysis of a selected cohort of patients with biochemical recurrence has been performed to explore potential predictors of PSMA PET positivity. We observed that pT3a or higher pathological status after radical prostatectomy, and high levels of PSA at the time of scan are significantly correlated with PSMA PET positivity. These findings may contribute to an optimization in the management of biochemical recurrence patients, via a better patient selection and imaging timing.

**Abstract:**

Localized prostate cancer (PCa) can be treated with radical prostatectomy (RP). Up to 30% of patients undergoing this procedure experience biochemical recurrence (BCR), namely the rise in serum prostate-specific antigen (PSA) levels during the post-surgical follow-up, requiring further treatments and with the risk of severe disease progression. Currently, the most accurate imaging technique to confirm, detect, and locate disease relapses in BCR patients is prostate-specific membrane antigen (PSMA)-targeted PET, as recommended by international clinical guidelines. The aim of the study was to investigate potential clinical and pathological predictors of PSMA PET positivity, validated by clinical and instrumental follow-up or histopathological data. In this study, a selected cohort of BCR patients after RP and no other PCa-related therapy who underwent either PSMA PET/CT or PSMA PET/MRI has been analysed. Among the considered predictors, both pathological staging after RP equal or higher than pT3a and higher PSA levels at the time of the scan were significantly correlated with PSMA PET positivity on multivariate logistic regression analysis. As expected, PSMA PET confirmed its role as an accurate imaging technique in the setting of BCR in PCa. These findings may inform appropriate and tailored patient selection and scan timing to optimize and fully exploit this powerful diagnostic tool.

## 1. Introduction

Despite advances in primary intended curative therapy for prostate cancer (PCa), 27–53% of patients develop a biochemical recurrence (BCR), with higher percentages depending on the preoperative risk and stage of cancer [1,2]. 

Patients showing BCR of PCa have a higher risk of developing distant metastases and PCa-related death [3,4]. Several clinical parameters including serum prostate-specific antigen (PSA) levels, PSA kinetics, and the International Society of Urological Pathology (ISUP) score have been shown to predict the risk of PCa recurrence after RP [5,6,7,8]. 

The identification of the possible sites of recurrence is therefore of paramount importance for decision-making on subsequent salvage management, in order to address patients to directed therapies with prolonged intervals of cancer-free survival [9,10]. 

In this scenario, imaging plays a key role in identifying local or distant recurrence when the PSA level is rising.

The significant relevance of an accurate imaging modality able to provide early and precise identification of the site of disease recurrence is highlighted by recent reports showing that a metastasis-directed approach may be suitable for patients with the intent of delaying clinical progression and eventually postponing the initiation of ADT and its related toxicity [11].

In fact, as patients in the early stages of BCR are still curable, the capability of imaging to precisely identify sites of recurrence is pivotal to choose the best therapeutic approach (e.g., salvage radiation therapy, metastases-directed therapy, and hormonal therapy). Positron emission tomography (PET) has gained particular attention for the restaging of PCa due to the introduction of new radiotracers, such as [^68^Ga]Ga-PSMA-11, that have better sensitivity and specificity for the detection of metastatic disease, compared to conventional imaging, also at low PSA levels [12,13,14]. 

EAU-EANM-ESTRO-ESUR-SIOG guidelines recommend performing PSMA PET after RP if the PSA level is >0.2 ng/mL [15]. However, patients experiencing BCR are a heterogeneous group, comprising individuals presenting first-time PSA relapse, others that already underwent salvage radiotherapy, hormone-naïve or receiving ADT, or even a combination of treatments. Yet, neither international guidelines nor most published studies investigating the predictors of PSMA PET positivity for the detection of recurrent PCa account for this heterogeneity.

Considering the heterogeneous nature of BCR could potentially result in more accurate and personalized guidelines for [^68^Ga]Ga-PSMA-11 PET execution in recurrent PCa. This, in turn, would ensue an earlier detection of the prostatic disease allowing prompt intervention with curative intent, ameliorating patients’ prognosis. 

Currently available literature investigating the role of PSMA PET in biochemical recurrent prostate cancer is highly heterogeneous regarding patients’ characteristics, as they include subjects treated with either surgery or radiotherapy for primary PCa, and both patients with and without hormonal therapy during biochemical recurrence.

The aim of this study is to identify clinical and pathological factors predicting [^68^Ga]Ga-PSMA-11 PET findings in PCa patients treated exclusively with RP and showing rising levels of PSA. 

## 2. Materials and Methods

### 2.1. Patients

A total of 253 PCa patients referred to our Institution for [^68^Ga]Ga-PSMA-11 PET because of rising levels of PSA after RP, from June 2020 to February 2022, were retrospectively considered for this study. Inclusion criteria were (1) previous treatment with RP and pelvic lymphadenectomy, (2) rising levels of PSA and (3) availability of all clinical and pathological variables to be considered in this study (Table 1). Exclusion criteria were (1) persistent disease defined as PSA ≥ 0.2 ng/mL after surgery and (2) any other PCa-related treatment prior, concomitant, or after RP. Eighty patients met the inclusion criteria and were considered for analysis.

### 2.2. PET Acquisition Protocol and Image Interpretation

PET scans were acquired using either Signa PET/MRI 3 Tesla system (GE Healthcare, Waukesha, WI, USA; N = 39) or PET/CT systems (Discovery-STE or Discovery-690, GE Healthcare, Waukesha, WI, USA; N = 41). [^68^Ga]Ga-PSMA-11 PET/MRI scans were acquired according to a previously published protocol [16]. PET/CT scans were acquired according to the joint EANM and SNMMI procedure guidelines for PCa imaging [17]. [^68^Ga]Ga-PSMA-11 PET image read-out was performed by two Nuclear Medicine Physicians with knowledge of all the available patients’ clinical and imaging information on the Advantage Workstation (AW, General Electric Healthcare, Waukesha, WI, USA), on which PET, MRI, CT, and fused images could be visualized in axial, coronal, and sagittal planes. In the event of disagreement or uncertain findings, the images were re-examined, and a consensus was reached. The whole-body distribution pattern of [^68^Ga]Ga-PSMA-11 was qualitatively assessed, and the presence of increased uptake deviating from the physiological distribution of the tracer was considered positive for malignancy [18]. The anatomical sites were defined based on MR or CT images co-registered with PET examinations.

### 2.3. Validation of [^68^Ga]Ga-PSMA-11 PET Findings

In all the cases when a clinical or instrumental follow-up was available, PET findings were validated by using a composite reference standard. In particular, [^68^Ga]Ga-PSMA-11 PET findings were considered true positive when at least one of the following criteria was met: (1) histological confirmation on surgically resected specimen; (2) progression (increase in number of pathological [^68^Ga]Ga-PSMA-11 uptake sites or increase in uptake intensity) on follow-up PET/CT or PET/MR studies associated with an increase in PSA level; (3) confirmation on conventional imaging either at baseline (including the diagnostic MR exam performed simultaneously to the [^68^Ga]Ga-PSMA-11 scan for patients examined with the fully hybrid 3 Tesla PET/MR scanner) or during follow-up; (4) disappearance or considerable reduction (i.e., number and intensity) in the [^68^Ga]Ga-PSMA-11 uptake on follow-up PET/CT or PET/MR scans after local or systemic treatment associated with a decrease in PSA level greater than 50%; (5) a decrease in PSA level greater than 50% after selective irradiation of the site of pathological [^68^Ga]Ga-PSMA-11 uptake. Patients with negative [^68^Ga]Ga-PSMA-11 PET were considered true negative in the absence of evidence of disease on conventional imaging or [^68^Ga]Ga-PSMA-11 PET/CT or PET/MR acquired during the follow-up period (median follow-up duration for both positive and negative findings: 18.6 months, range: 8–28.5 months).

### 2.4. Statistical Analysis

All statistical analyses were performed with R statistical software v4.0.5 (R Core Team (2021), R Foundation for Statistical Computing, Vienna, Austria). The variables investigated in this study were: (1) the ISUP score, dichotomized as ISUP ≥ 4 or <4; (2) pT stage, as pT ≥ 3a or pT < 3a; (3) PSA at the time of the scan, divided in PSA ≤ 0.5, between 0.5 and 1, and PSA ≥ 1 ng/mL; (4) presence of positive surgical margins; and (5) lymph nodal involvement at histopathological analysis. Chi-squared, whenever feasible, or Fisher’s exact test was used to correlate clinical and histopathological data with PET findings (PET positive vs. negative) on a patient basis. Univariate and multivariate binary logistic regression was performed to assess the factors predicting positive [^68^Ga]Ga-PSMA-11 PET. *p* < 0.05 was considered statistically significant.

## 3. Results

### 3.1. Patients

Eighty patients (mean age: 69.5 years, SD: 6.8) with a median PSA at the time of the scan of 0.345 ng/mL (range: 0.01–5.24 ng/mL) were retrospectively enrolled in this study. All patients underwent [^68^Ga]Ga-PSMA-11 PET (15 Discovery-STE PET/CT, 26 Discovery-690 PET/CT, and 39 SIGNA PET/MRI) for biochemical recurrence of PCa after radical prostatectomy. Patients’ characteristics are reported in Table 1.

### 3.2. PET Findings

**Figure 1:**
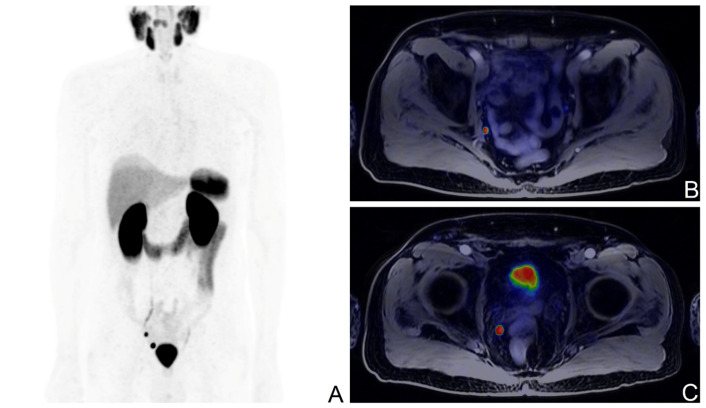
[^68^Ga]Ga-PSMA-11 PET/MR scan of a BCR patient with the ISUP score of 3, pT3b after RP; PSA levels at the time of the scan: 0.14 ng/mL; Maximum Intensity Projection image (**A**), fused axial PET/MR views showing a pathological right iliac lymph node (**B**), and a pathological right pararectal lymph node (**C**).

**Figure 2:**
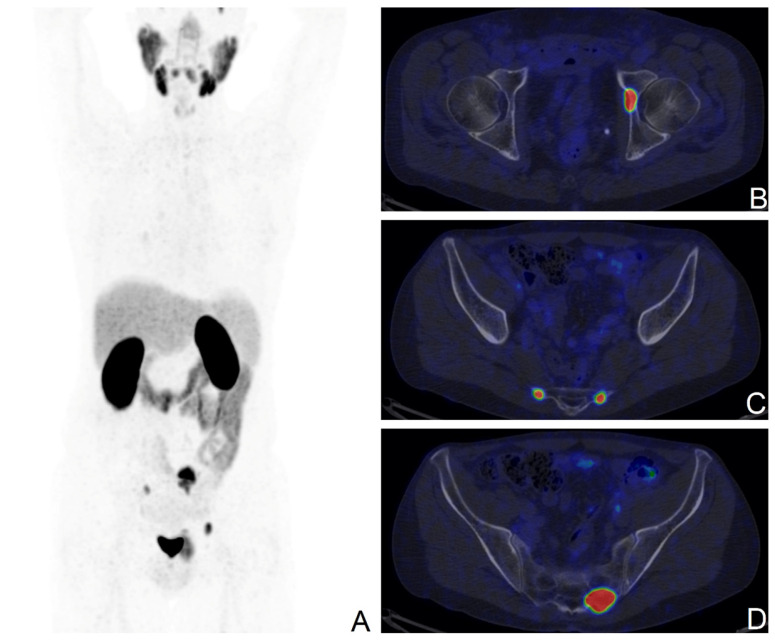
[^68^Ga]Ga-PSMA-11 PET/CT scan of a BCR patient with the ISUP score of 5, pT3a after RP; PSA levels at the time of the scan: 1.43 ng/mL; Maximum Intensity Projection image (**A**), fused axial PET/CT views showing secondary bone lesions at the left acetabulum (**B**), and at the sacrum bilaterally (**C**,**D**).

[^68^Ga]Ga-PSMA-11 PET was positive in 39/80 patients (median PSA: 0.43 ng/mL, range: 0.13–4.66) and negative in 41/80 (median PSA: 0.3 ng/mL, range: 0.01–5.22). On a regional basis, [^68^Ga]Ga-PSMA-11 uptake was observed in correspondence of the prostatic fossa in 6/39 patients (15%), of lymph nodes in 27/39 patients (69%), of bone in 12/39 patients (31%), and in the lungs in 1/39 patients. Among the 27 patients with lymph-nodal [^68^Ga]Ga-PSMA-11 uptake, 20/27 showed involvement of regional lymph nodes only, 4/27 solely non-regional lymph nodes, and in 3/27 [^68^Ga]Ga-PSMA-11 uptake was present in both regional and non-regional lymph nodes. Five patients with PSA < 0.2 ng/mL showed pathological findings at PET, all of them presenting pT ≥ 3a (Figure 1 and Figure 2).

### 3.3. Validation of [^68^Ga]Ga-PSMA-11 PET Findings

Clinical or instrumental follow-up was available for 59/80 patients. A total of 21/59 findings were confirmed to be true positive at follow-up examination, while the positive findings at [^68^Ga]Ga-PSMA-11 PET were not supported by further evidence in 5/59 patients. Evidence of recurrent PCa missed at PET was identified in 9/80 patients. Finally, 24 patients that were negative at [^68^Ga]Ga-PSMA-11 PET did not show any evidence of recurrent disease in the follow-up period. This resulted in an accuracy = 0.76, sensitivity = 0.7, specificity = 0.83, positive predictive value = 0.81, and negative predictive value = 0.73.

### 3.4. Predictors of [^68^Ga]Ga-PSMA-11 PET Positivity

Patients with the ISUP score ≥ 4, or pT ≥ 3a, or higher PSA levels were more likely to be [^68^Ga]Ga-PSMA-11 PET positive (X2 *p* = 0.037, 0.023 for ISUP and pT stage; and Fisher *p* = 0.005 for PSA), while presence of positive surgical margins and lymph node involvement at prostatectomy were not associated with [^68^Ga]Ga-PSMA-11 PET detection rate (*p* > 0.05) (Table 2). In the univariate logistic regression, ISUP score, pT stage, and PSA levels were significantly associated with an increased risk of positive [^68^Ga]Ga-PSMA-11 PET. However, the higher risk of positive PET was confirmed only for patients with pT ≥ 3a and more elevated PSA levels in the multivariate analysis (OR = 3.53 and 3.18, *p* = 0.021 and 0.004; respectively). Conversely, the effect of the ISUP score was no longer significant when analysing all variables together (Table 3).

## 4. Discussion

In the last years, PSMA PET has established its role as the most accurate imaging technique available in the clinical scenario of BCR in PCa, and it is included in the current guidelines [15]. In particular, the superiority of PSMA-targeted imaging over Choline PET, the former advanced diagnostic tool of choice, has been largely demonstrated, especially at low PSA levels and in the detection of nodal disease [19,20].

In the present study, including treatment-naïve patients with BCR after RP, we determined that the strongest predictor of PSMA PET positivity is represented by serum PSA levels ≥ 1 ng/mL. This result is consistent with the currently available literature, which reports that the proportion of positive [^68^Ga]Ga-PSMA-11 PET scans rises alongside PSA values [21]. 

Several studies have been in support of this correlation between PSA values and PSMA PET positivity, although it is worth noting that the clinical scenario of BCR is heterogeneous. In fact, the majority of the available data have been pooled by heterogeneous cohorts, including patients treated with surgery or radiotherapy or combined approaches. Additionally, the possible interference of previous or ongoing medical therapy and the risk profile of the selected population are definitely issues that need to be taken into account. Our study, focusing exclusively on patients with no other PCa-related treatment other than RP, aims to contribute to the already solid body of literature available on the topic, providing consistent data extracted by an especially homogeneous cohort, and minimising interfering factors.

Fendler et al., in their compelling prospective work, reported higher levels of sensitivity and specificity compared to our results; however, their cohort included patients who underwent different combinations of primary treatments and presented higher-risk diseases in terms of PSA levels (median of 2.1 ng/mL, versus 0.345 ng/mL in our paper) [13]. [^68^Ga]Ga-PSMA-11 PET detection rate was identical in patients with PSA lower than 0.5 ng/mL (38%), and consonant with levels between 0.5 ng/mL and 1 ng/mL (57% vs. 64%). In a similar clinical scenario, Caroli et al. prospectively analysed data from biochemically recurrent patients after either RP or radiation therapy [22]. Their cohort was more comparable to the one analysed in the present work in terms of PSA levels distribution, with a median at the time of the scan of 0.83 ng/mL, and they achieved analogous levels of detection rate. Accordingly, in several papers evaluating BCR patients after RP, radiation treatment, or both, it is possible to infer the diagnostic performance of PSMA PET in patients treated with exclusive surgery only. Afshar-Oromieh et al., in their seminal 2015 paper, evaluated a large yet heterogeneous cohort of PCa patients, identifying an increasing growing probability of [^68^Ga]Ga-PSMA-11 PET positivity with the rise in PSA levels (47.1% at PSA values below 0.2 ng/mL, 50% between 0.21 and 0.5 ng/mL, 58.3% between 0.51 and 1 ng/mL, and 71.8% between 1.1 and 2 ng/mL) [23]. Meredith et al. investigated BCR patients treated with both RP and radiation therapy only, and in the surgical subgroup, the detection rates of [^68^Ga]Ga-PSMA-11 PET at different PSA levels are similar to what observed in our cohort (namely, 11.3% at <0.2 ng/mL, 26.6% at 0.2 to 0.5 ng/mL, 53.3% at 0.5 to 1 ng/mL, 79.1% at 1 to 2 ng/mL, and 95.5% at ≥2 ng/mL) [24]. The paper from Habl et al. focused on the role of [^68^Ga]Ga-PSMA-11 PET in radiation treatment planning in BCR patients after RP, and in their sub-cohort of patients treated with surgery, [^68^Ga]Ga-PSMA-11 PET was positive in 71% of patients; this higher proportion of positive scans reflects the higher biochemical risk profile of this population compared to the one of the present work (median PSA level of 0.81 ng/mL vs. 0.345 ng/mL) [25]. These results are in line, and they all confirm the accuracy of the technique and the strong predictive value of PSA levels at the time of the scan. 

Several studies have presented similar designs and selection process to the present manuscript, focusing mainly or solely on patients who underwent RP and no other oncological treatment [26,27,28,29,30]. As expected, the global PET detection rates vary according to median PSA values in the examined populations, but results in sub-cohorts of patients with similar biochemical risk profiles are consistent altogether and with what was observed in our paper.

Another major finding of the present work is that pT staging ≥ 3a after RP emerged as a significant predictor of [^68^Ga]Ga-PSMA-11 PET positivity. This may reflect the difficulty in the radical surgical removal of microscopic disease in locally complex scenarios and the biological aggressiveness of these cancers. It is worth noting that, in our cohort, all the five patients displaying a positive [^68^Ga]Ga-PSMA-11 scan performed with serum PSA levels below 0.2 ng/mL, lower than the value recommended by current guidelines, presented in fact a staging of pT3a or higher. 

Similarly, Caroli et al. explicitly evaluated the potential predictive value of pT staging [22]. The group identified a consistent trend by dichotomizing patients with the same threshold of pT3a, although they did not reach statistical significance (*p* = 0.07).

We found that the ISUP score ≥ 4 is associated with a higher probability of PET positivity on univariate logistic regression analysis, although this trend was not confirmed on multivariate logistic regression. This result is consistent with the available literature, as other groups have explored this and other possible clinical predictors such as positivity of examined surgical margins and lymph node pathological staging at surgery, without obtaining consistent significant results.

In a recent study performed by Bianchi et al., an external validation of a nomogram predictive for PSMA PET positivity was conducted in 1639 patients with recurrent prostate cancer [31]. This large cohort of patients included heterogeneous clinical conditions, with subjects experiencing first BR, PSA relapse after salvage therapy, PSA persistence after radical prostatectomy, and advanced-stage PCa patients after second-line systemic therapies. Conversely, in the present study, a very homogeneous cohort of patients was considered to explore possible predictors of positivity of PSMA PET, including only patients experiencing biochemical recurrence after radical prostatectomy, with no other concomitant treatment between prostatectomy, biochemical recurrence, and PSMA PET. 

Some limitations of the present study should be outlined. It is retrospective and monocentric, suffering from typical limitations such as selection biases and patient referral biases. It is also worth noting that the willfully strict patient selection strategy certainly comes with strengths and weaknesses. Data provided from this highly homogeneous cohort can be regarded as valuably free from additional factors influencing findings and analyses. On the other hand, this factor demands further inquiries to extend our results in BCR patients undergoing medical therapy, and in patients treated with external beam radiation therapy—where cT staging as established on imaging assessments may serve as a surrogate to pT staging predictive role.

PSMA PET is by far the most powerful diagnostic tool available to detect BCR and inform subsequent patient management, as concurred by international guidelines [11]. The identification of strong clinical predictors of [^68^Ga]Ga-PSMA-11 PET positivity may further improve the cost-efficiency and the diagnostic prowess of this technique, by avoiding unnecessary exams and expenditures in low-risk patients, and conversely by anticipating diagnosis in high-risk ones. In the era of precision and personalised medicine, we should strive to refine our strategy on when and whether to perform this accurate yet expensive exam, to the benefit of all prostate cancer patients. In treatment-naïve patients after RP, a pathological staging pf pT3a or higher may add value to the currently recommended scan timing strategy based on PSA levels alone, by anticipating PET scan in high-risk patients even with PSA serum values lower than 0.2 ng/mL. Prospective and larger data are needed to confirm these results, with the goal to inform more tailored clinical strategies.

## 5. Conclusions

In a very homogeneous clinical setting of BCR after RP, without any other treatment between prostatectomy, biochemical recurrence, and PSMA PET, both pre-scan PSA serum levels and pT staging at RP are strong predictors of [^68^Ga]Ga-PSMA-11 PET positivity. Considering both easily available clinical data in combination could help overcome a dichotomic, one-size-fits-all approach to inform tailored [^68^Ga]Ga-PSMA-11 PET timings. These personalised strategies could achieve earlier diagnoses and avoid unnecessary exams.

## Figures and Tables

**Table 1:** Patients’characteristics.

Characteristic	Value
N	80
Age (years)	
Mean ± SD	69.5 ± 6.8
Range	53–86
ISUP score	
ISUP < 4	55
ISUP ≥ 4	25
Pathological T stage	
pT < 3a	36
pT ≥ 3a	44
PSA at the time of the scan (ng/mL)	
PSA ≤ 0.5	56
PSA between 0.5 and 1	11
PSA ≥ 1	13
Positive surgical margins	
No	67
Yes	13
Lymph node involvement	
No	69
Yes	11

**Table 2:** Association between clinical parameters and [^68^Ga]Ga-PSMA-11 PET positivity.

Stratification	No. Patients	Positive Results, No. (%)	*p* Value
ISUP score			
ISUP < 4	55	22 (40%)	0.037
ISUP ≥ 4	25	17 (68%)	
pT stage			
pT < 3a	36	12 (33%)	0.023
pT ≥ 3a	44	27 (61%)	
PSA (ng/mL)			
PSA ≤ 0.5	56	21 (38%)	0.005
PSA 0.5–1	11	7 (64%)	
PSA ≥ 1	13	11 (85%)	
Positive surgical margins			
No	67	33 (49%)	1
Yes	13	6 (46%)	
Lymph node involvement			
No	69	32 (46%)	0.343
Yes	11	7 (64%)	

**Table 3:** Univariate and multivariate binary logistic analysis of factors predicting positive [^68^Ga]Ga-PSMA-11 PET.

	Univariate Logistic Regression		Multivariate Logistic Regression	
Parameters	OR	*p*	OR	*p*
ISUP score	3.16	**0.023**	1.63	0.397
pT stage	3.16	**0.014**	3.53	**0.021**
PSA (ng/mL)	3	**0.002**	3.18	**0.004**
Positive surgical margins	0.88	0.838	1.26	0.736
Lymph nodes	2.02	0.294	1.71	0.455

## Data Availability

All data needed to replicate our analyses are available upon request from the corresponding author.
